# Indirect Virus Transmission via Fomites Can Counteract Lock-Down Effectiveness

**DOI:** 10.3390/ijerph192114011

**Published:** 2022-10-27

**Authors:** Torsten Thalheim, Tyll Krüger, Jörg Galle

**Affiliations:** 1 Interdisciplinary Centre for Bioinformatics (IZBI), Leipzig University, Haertelstr. 16-18, 04107 Leipzig, Germany; 2 Institute of Computer Engineering, Control and Robotics, Wroclaw University of Science and Technology, Janiszewskiego 11-17, 50-372 Wrocław, Poland

**Keywords:** virus transmission, contact tracing, lock-down, modelling epidemics, SEIR, agent-based model

## Abstract

**Highlights:**

**What are the main findings?**

- Contact tracing (CT) alone can control epidemic spreading
- CT efficacy changes under mobility lockdowns (LDs)
- A small fraction of indirect transmission can impede disease control

**What is the implication of the main finding?**

- Detailed knowledge regarding transmission routes is crucial to determine efficient non-pharmaceutical intervention strategies
- Reduction of indirect transmission via fomites becomes particular important in the course of mobility LDs

**Abstract:**

The spread of severe acute respiratory syndrome-coronavirus-2 (SARS-CoV-2) has raised major health policy questions. Direct transmission via respiratory droplets seems to be the dominant route of its transmission. However, indirect transmission via shared contact of contaminated objects may also occur. The contribution of each transmission route to epidemic spread might change during lock-down scenarios. Here, we simulate viral spread of an abstract epidemic considering both routes of transmission by use of a stochastic, agent-based SEIR model. We show that efficient contact tracing (CT) at a high level of incidence can stabilize daily cases independently of the transmission route long before effects of herd immunity become relevant. CT efficacy depends on the fraction of cases that do not show symptoms. Combining CT with lock-down scenarios that reduce agent mobility lowers the incidence for exclusive direct transmission scenarios and can even eradicate the epidemic. However, even for small fractions of indirect transmission, such lockdowns can impede CT efficacy and increase case numbers. These counterproductive effects can be reduced by applying measures that favor distancing over reduced mobility. In summary, we show that the efficacy of lock-downs depends on the transmission route. Our results point to the particular importance of hygiene measures during mobility lock-downs.

## 1. Introduction

In 2020, governments attempted to control the COVID-19 pandemic with non-pharmaceutical interventions ranging from limiting gathering sizes, closures of business and educational institutions, to stay-at-home orders, each of which reduced virus transmission with different efficacy [1]. Given the impact of these interventions on resources and individual liberty, they can be applied for a limited period only. Thus, additional control and monitoring strategies have been recommended. One key strategy is contact tracing (CT), i.e., identifying and monitoring people who have been in close contact with individuals with confirmed diagnoses.

Close contact with infectious individuals, in particular long-term, face-to-face contact, is commonly accepted as the main transmission route of SARS-CoV-2. In contrast, transmission via contaminated, highly frequented areas is considered to be of minor importance, in particular in community settings [2,3]. It has been shown that stay-at-home mandates reduce disease spreading [4], supporting that infection with SARS-CoV-2 is mainly driven by direct contact transmission. However, we show that mobility lockdowns can decrease CT efficacy and thereby may counteract disease control.

A prerequisite of such effects is a small contribution (~10%) of indirect virus transmission. Indirect transmission is known to be important for other corona viruses such as SARS and MERS [5]. Indeed, SARS-CoV-2 can remain viable on non-adherent surfaces for several days, as reviewed in [6,7], or even longer on personal protective equipment such as gloves, coveralls or face masks [8]. This time is long enough to enable viral spreading, though there is evidence that sunlight rapidly inactivates the virus [9]. Thus, indirect transmission may occur preferentially indoors via high-touch surfaces as observed in Guangzhou, e.g., [10]. In the UK, the contribution of indirect transmission routes via fomites to COVID-19 death cases has been estimated to reach 25% [11].

Mathematical models have been applied to study the conditions necessary for efficient CT [12,13,14,15], the impact of supportive techniques [16], and their potential to allow re-opening [17]. These studies use different modeling strategies such as stochastic or deterministic branching models and agent-based models, and they can cover a broad range of spatial details [17]. However, all of these studies assume direct (contact-based) virus transmission. In fact, indirect transmission scenarios were modeled long before the COVID-19 pandemic [18] and even consider the effects of preferential attachment [19]. To our knowledge, such models have not yet been investigated with respect to non-pharmaceutical interventions.

Here, we use an agent-based model to simulate epidemic spread in scenarios that consider both direct and indirect infection routes. We simulate spreading with and without CT and study lock-down (LD) scenarios for both transmission types. Thereby, we provide insight into mechanisms that might limit or even counteract epidemic control by CT. It was neither our objective to provide a quantitative model of the early COVID-19 epidemic nor to simulate the impact of particular travel modes on epidemic spread. We focus on the interplay between different control strategies and show that they can counteract each other. By using model parameters of the simulated epidemic similar to those of the COVID-19 epidemic, we demonstrate that such interactions can become relevant under real conditions, which requires better characterization of transmission routes in general. We expect our study to contribute to a better understanding of potential spreading dynamics.

## 2. Methods

Our modelling approach builds on the so-called SEIR model. Accordingly, we divide the population (*N*) into four different subpopulations: (i) individuals that are healthy but susceptible to the infection (*S*), (ii) newly infected individuals that are not infectious, called “exposed” (*E*), (iii) infectious individuals (*I*), and (iv) those individuals who recovered from the disease (*R*). Thus, it yields: *S* + *E* + *I* + *R* = *N*. Agents cannot become infected after they have recovered. We neglect births and deaths of agents. In a continuum approach, this model is described by four differential equations:

(1) $$\begin{array}{cc} \mathrm{Susceptible}:\;\frac{dS}{dt}=-\beta \frac{S}{N}I & \;\mathrm{Exposed}:\;\frac{dE}{dt}=\beta \frac{S}{N}I-\alpha E\\ \mathrm{Infected}:\;\frac{dI}{dt}=\alpha E-\gamma I & \;\mathrm{Recovered}:\frac{dR}{dt}=\gamma I \end{array}$$

Here, *t* is the time, *β* the transmission rate, *α* the conversion rate and *γ* the recovery rate. The parameters *β* and *γ* can be estimated based on epidemic spread. For the number of cumulative cases *P* yields *P = N − S.* Thus, an increase of the daily cases Δ*P* with the number of currently infectious individuals *I* provides an estimate for *β* in the initial phase of the epidemic (*S* ≈ *N*). The change of the daily recovered cases Δ*R* with *I* provides the recovery rate *γ* [20]. The model parameter are summarized in Table 1.

Assuming that the daily cases and daily recovered cases seen in an epidemic are proportional to Δ*P* and Δ*R*, respectively, one can estimate *β* and *γ* from public data. In our study, in order to model realistic epidemic spreading dynamics, we use data on the COVID-19 epidemic spread in Austria, Germany and Switzerland in the first epidemic wave in the summer of 2020. They are very similar and suggest average values *β^ex^* = 0.25/day and *γ^ex^* = 0.05/day (Figure S1, Supplementary Materials). These values are in agreement with an estimate of the parameters for Austria by Bayesian analysis [21] and provide an estimate of the basic reproduction number: *R*_0_ = *β*/*γ* = 5.

**Figure 1:**
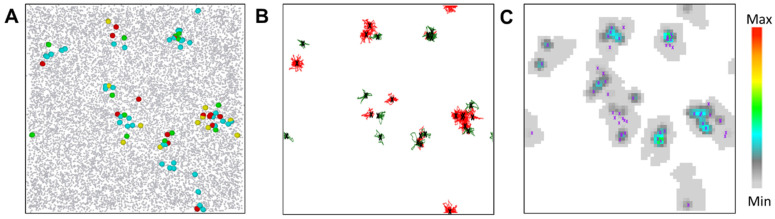
Agent properties. (**A**) The spatial distribution of agents (16,000 agents) at *t*_1_ = 4 weeks. Color encodes the type of agent. Grey: *S*, yellow: *E*, red: *I_s_*, green: *I_a_*, cyan: *R*. The size of agents in state *E*, *I_s_*, *I_a_* and *R* is enhanced for better identification. (**B**) The traces of agents during their infectious state, and (**C**) the viral load *V_L_* induced by these agents. Small crosses indicate the agent’s base position.

In our study, we use a stochastic realization of the SEIR-model with discrete time steps Δ*t*. A summary of the model design can be found in the Supplementary Materials (Text S2). In the reference system, we consider *N_ref_* ≈ 16,000 agents (Figure 1A) that perform a truncated random walk on a quadratic area *A* with periodic boundary conditions (Figure 1B). The jump rate is constant (1/Δ*t*) and the step size in both the x- and y-direction is equally distributed between 0 and *r_max_*. We assign each agent an individual base position (*p* Є *A*), to which it returns repeatedly after *t_ret_* = 12 h in a single jump [22]. We do not consider heterogeneity of agent mobility, household structures and do not model travel.

For the duration of the exposed state, *T_E_* = 1/*α*, we assume two days [23]. In contrast to the classical SEIR model, we do not vary this time throughout the simulation; thus, the transition from exposed to infectious (*E* to *I*) is deterministic. Other agent properties also differ from the classical SEIR-model: The number of infectious agents (*I*) is randomly split into two populations, symptomatic (*I_s_*) and asymptomatic (*I_a_*) agents. While symptomatic infectious agents are always detected after the incubation time, asymptomatic infectious agents are not detectable without testing (see below). The average fraction of asymptomatic cases: *F_A_* = *I_a_*/(*I_s_* + *I_a_*) is fixed. We assume a minimal duration of the infectious state *T_I_* before an agent can recover. This is an option to model data on the daily recovered cases that show separate branches for increasing and decreasing numbers *I* = (*I_s_* + *I_a_*) (Figure S2A, Supplementary Materials). After that time span, individuals can recover with the transition probability *Γ*Δ*t* per time step. We consider two different transmission scenarios:

**Table 1:** Reference model parameter (*N_ref_* ≈ 16,000, *A* = 1.6 km^2^). For simplification, we consider fixed parameters (*T_E_*, *T_I_* and *T_N_*) although they might be broadly distributed (see [24]).

Parameter	Symbol	Value	Comment
duration of the exposed state	T_E_ = 1/*α*	2 days	[23], 2 days
incubation time	*T_N_*	5 days	[25], 4.75 days
minimal time of infectious state	*T_I_*	5 days	[26], *T_N_* − *T_E_* + 2 days
fraction of asymptotic cases	*F_A_*	1/2, 2/3,…, 5/6	serological studies, see text
agent return time	*t_ret_*	12 h	[22], 12 and 24 h
time step	Δ*t*	3.6 min	*t_ret_*/200
maximum step size: reference LD20	*r_max_*	3.0 m 0.6 m	average distance from base position after *t_ret_*: ~30 m ~6 m
contact/infection radius	*r_in_*	4 m	set
rate of direct transmission rate of indirect transmission	*β_di_* *β_in_*	0.2/day 0.2/day	transmission rate close to *β^ex^* (±10%, Figure S1) set
number of interaction points contamination rate per agent virus load half-life-time	*M* *a_L_* *d_L_*	8450 100/*t_ret_* 15 h	set set transmission rate close to *β^ex^* (±10%)
recovery rate after *T_I_*	*γ*	0.05/day	recovery rate ≈ *γ^ex^* (Figure S1)

**Direct transmission**: Infectious agents can infect susceptible agents with a constant transmission rate, *β_di_*, if they are in contact. To calculate contact events, we assign each agent a contact radius *r_in_*. If two agents come closer than 2*r_in_*, they are considered to have contact as long as their distance remains smaller than 2*r_in_*. Contact is tested in each time step Δ*t*. The expected number of infections per infectious *i* per day, *w_di_*, is accordingly given by *β_di_*∑*_j_*(*k_j_*Δ*t*), where (*k_j_*Δ*t*) is the duration of the contact *j* (*k_j_* time steps). The sum runs, over all, potentially multiple contacts with susceptible agents within a day (2*t_ret_*). The *β_di_* was chosen such that *w_di_*/(2*t_ret_*) within the first weeks is close to the observed values of *β^ex^* (±10%) in the COVID-19 epidemic.

**Indirect transmission:** Infectious agents leave a trace of virus behind. They contaminate the environment, represented by *M* interaction points randomly distributed over *A*. Each point *j*, thus, has a dimensionless virus load *V_L_*(*j*, *t*) (Figure 1C). This load increases during the contact time with an infected agent in every time step by *a_L_* Δ*t*/N_c_ (*a_L_*: virus contamination parameter, *N_c_*: number of contact points of the agent) and decreases with a half-life-time *d_L_* of about 15 h. Susceptible agents in contact with a contaminated point *j* become infected at the rate $\;\beta_{in}V_{L}\left(j,t\right)$. The expected number of infections per infectious *i* per day, *w_in_*, is given by $\beta_{in}\underset{j}{\sum }\underset{k}{\sum }\left(V_{L}^{i}\left(j,t\right)\Delta t\right)$, where *j* runs over all contacts by susceptible agents with points *j* contaminated by *i* within a day, and *k* runs over the time steps of the individual contact. $V_{L}^{i}\left(j,t\right)$ is the load at point *j* left behind by *i.* To facilitate comparison between direct and indirect transmission scenarios, the half-life-time *d_L_* was adjusted such that *w_in_*/(2*t_ret_*) in the first weeks is again close to observed values of the COVID-19 epidemic of *β^ex^* (±10%). For mobile agents, the number of agents contacting the points contaminated by an infectious agent is much higher than the number of agents contacting this agent directly. Thus, the risk of an indirect infection becomes relevant, although the risk per individual surface contact may be small.

**Modeling quarantine and CT:** In our simulations, exposed cases (*E*) are not detectable. Symptomatic infectious agents (*I_s_*) always become detected after an incubation/detection time *T_N_*, which is assumed to be longer than the duration of the exposed state *T_E_*. Thus, virus transmission is possible for these agents. They are put under quarantine (no further contacts) immediately after detection until they recover (*R*). We refer to this as a “quarantine scenario”. Asymptomatic infectious agents (*I_a_*) are not detectable without testing; thus, without CT, virus transmission occurs during the infection time. Serological studies in Germany provided numbers for the fraction of “not detected cases” of about 0.75–0.80 in hotspots [27,28]. Theoretical estimations suggest values of *F_A_* = 0.56 for several European countries [29]. Here, we use *F_A_* = 0.50 as a reference value. If CT is utilized, all agents that have had contact with an identified symptomatic infectious case in the last week are tested for infection. Detected cases are put immediately under quarantine, but their contacts are not traced further. Test efficacy of PCR tests has improved and error rates as high as in the early phase [30] are unlikely. However, most of the tests are still far from providing 100% sensitivity and/or specificity [31,32]. Nevertheless, we consider an ideal test as identifying infected cases without error.

**Modeling lockdowns and physical distancing:** To gain insight into the relationship between CT performance and agent mobility, we simulate mobility lockdowns (LDs). In these simulations, we reduce the maximum step size of the truncated random walk to a fixed percentage of the reference value (for LD20 to 20%). Thereby, we maintain the jump rate and the rate of returning to the base position fixed, i.e., we maintain the basic pattern of mobility [22]. During a LD, the number of agent contacts decreases (i.e., the number of tests per detected case too), but the average time of the contacts increases. We do not model the effects of household quarantine. Thus, after detection of a case, the simple quarantine scenario is still applied. For comparison, we simulated scenarios of physical distancing as well. Details are described in the Supplementary Materials (Text S2).

## 3. Results

**Random jump model.** We started our study simulating a random jump model without indirect transmission (Ran). Here, each agent can reach each position within Δ*t* by a single jump. Thereby, the positions reached are uniformly distributed on the area A. We simulated the epidemic without symptomatic infections (*F_A_* = 1) for different numbers of agents *N*. Accordingly, infected agents are not detected. We started each simulation by infecting 10 randomly selected agents and followed the epidemic over at least 150 days. Results of 10 simulations were averaged.

The basic reproduction number of this scenario is given by:

(2) $$R_{0}\left(N\right)=R_{0}\left(N_{ref}\right)N/N_{ref}$$

Moreover, the maximum fraction of infected agents in the SEIR model is given by [33]:

(3) $$J_{\max}/N=\;\max\left(E+I_{s}+I_{a}\right)N^{-1}=1-\left(1+\ln(R_{0}\left(N\right)\right))R_{0}{\left(N\right)}^{-1}$$

**Figure 2:**
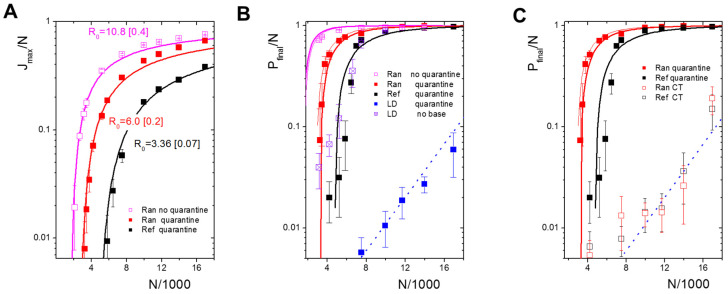
Characteristics of the epidemic for different agent numbers and mobility. (**A**) Maximum fraction of infected agents, $J_{max}/N$, for the random jump model (Ran) with *F_A_* = 1 (pink). Quarantine of symptomatic agents (*F_A_* = 0.5, red) and reduction of mobility (Ref, black) decrease the maximum for all *N*. Symbols are simulation results, lines represent the SEIR solutions with fitted *R*_0_. (**B**) Final size of the epidemic, $P_{final}/N$. Symbols are simulation results, solid lines represent the SEIR solutions with fitted *R*_0_ (thick) and analytical *R*_0_ (thin, for Ran results only). The blue dotted line is an exponential growth curve for the subcritical regime. Reference mobility (Ref) keeps the epidemic under-critical up to an agent number of about 5000. LD20 mobility (blue) extends the subcritical regime beyond *N_ref_*. Loss of the base position at LD20 mobility (violet) abrogates the LD effect. (**C**) Applying CT keeps the epidemic under-critical for *N < N_ref_*. Thus, it has a similar effect on $P_{final}/N$ as a LD20. Lines as in (**B**). All errors: sd.

Thus, $R_{0}\left(N_{ref}\right)$ can be estimated from simulation results fitting $J_{max}/N$. We obtained $R_{0}\left(N_{ref}\right)$ = 10.8 [0.4] for our parameter settings (Figure 2A). This is close to the analytical value $R_{0}\left(N_{ref}\right)\;\;$ = 10.05 (Text S4, Supplementary Materials). A further important property of the SEIR model, the final size of the epidemic $P_{final}/N$, i.e., the cumulative fraction of cases at infinite time, is given by the largest solution of the equation:

(4) $$\ln\left(1-P_{final}/N\right)+R_{0}\left(N\right)P_{final}/N=0.\;$$

These solutions describe the simulation results very well (Figure 2B). Having $R_{0}\left(N_{ref}\right),$ the rate of transmission $\beta_{1}^{Ran}$ for *F_A_* = 1 is given by $R_{0}\left(N_{ref}\right)/\left(T_{I}+\Gamma^{-1}\right)$. For $R_{0}\left(N_{ref}\right)$ = 10.8, one obtains $\beta_{1}^{Ran}$ = 0.43 [0.02]. This result for *F_A_* = 1 can be used to provide an approximation for $R_{0}\left(N_{ref}\right)$ if symptomatic infectious agents are present. For *F_A_* = 0.5, the reproduction number is given by $R_{0}\left(N_{ref}\right)=\beta_{1}^{Ran}\left(T_{I}+\Gamma^{-1}+T_{N}-T_{E}\right)/2$. Thus, one obtains $R_{0}\left(N_{ref}\right)$ = 6.0 [0.2].

Corresponding results for $J_{max}/N$ and $P_{final}/N$ (F_A_ = 0.5, Text S5, Supplementary Materials) nicely agree with our simulation results (Figure 2A,B). Note that in these, as in all of the following simulations, we assume that the infection of all initially infected agents cannot be detected, i.e., they all contribute to *I_a_*.

**Effects of confined mobility.** Our model assumes two limitations of agent mobility: a truncated jump size and a base position. In order to quantify their effects, we simulated limited mobility under the quarantine scenario (*F_A_* = 0.5). Reducing mobility to the reference scenario (Ref), i.e., assuming a maximum jump size *r_max_* = *r_ref_* and introducing a base position, reduces the value of $R_{0}\left(N_{ref}\right)$. Fitting *J_max_*/*N* using Equations (2) and (3), we observed $R_{0}\left(N_{ref}\right)$ = 3.36 [0.07] (Figure 2A) and $\beta_{0.5}^{Ref}\;$= 0.24 [0.02] (≈*β^ex^*). Thus, the number of agents at which the epidemic becomes over-critical $N_{ref}/R_{0}\left(N_{ref}\right)$ is slightly below 5000.

Locking down mobility by decreasing the maximum step size *r_max_* to 20% of the reference scenario (LD20) increases this number beyond *N_ref_*, i.e., results in $R_{0}\left(N_{ref}\right)$ < 1. A similar reduction of *R*_0_ (64–85% at *R*_0_ = 3.5) by LDs were observed in the COVID-19 epidemics [34]. Thus, we used it as a standard. For a subcritical epidemic, clusters of infected agents expand locally, but do not percolate. If percolation is reached, *P_final_*/*N* becomes largely independent of the mobility and follows Equation (4) (Figure 2B). Loss of the base position under LD20 has similar effects as increasing the maximum step size *r_max_* to the reference value *r_ref_* (Figure 2B).

The differences due to limited mobility largely vanish (Figure 2C) when CT is introduced; CT can control the epidemic. For the chosen parameters, it results in similar $P_{final}/N$ as for LD20 mobility, independent of the actual mobility. These effects are described in more detail in the following. For the reference parameters used in the following, the model describes a slightly overcritical epidemic, i.e., with an effective reproduction number *R_eff_* slightly above one. This simulates the situation reached under non-pharmaceutical interventions in many countries in the first COVID-19 wave [20].

**Effects of measures under different infection scenarios.** Next, we analyzed epidemic spread for hypothetical complete direct transmission and complete indirect transmission scenarios. Again, we started each simulation by infecting 10 randomly selected agents and assigned them to *I_a_*.

**Figure 3:**
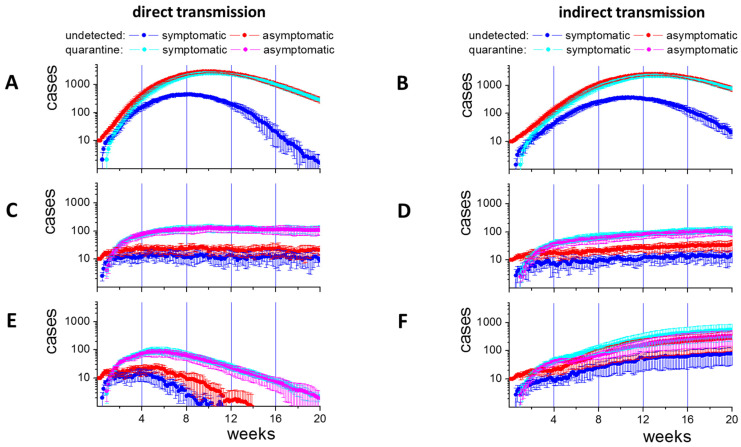
Epidemic spread for direct and indirect transmission. Time series of case numbers for controlled spreading without CT (**A**,**B**), with CT (**C**,**D**) and for LD after 4 weeks in parallel to CT (**E**,**F**). Case numbers are shown for detected and undetected symptomatic and asymptomatic cases (averages over 10 simulations, errors: sd). Without CT, case numbers peak between 8 and 13 weeks. With CT, they stabilize after short time at a much lower level. An LD20 is efficient for direct (*R_eff_* < 1) but counterproductive (*R_eff_* > 1) for indirect transmission. (greyscale version: see Supplementary Materials Figure S6).

Without CT, the epidemic with direct transmission reaches a maximum number of infected cases after about 10 weeks (Figure 3A). In the case of indirect transmission, the epidemic reaches a slightly lower maximum after about 13 weeks (Figure 3B).

For direct transmission, the epidemic can be efficiently curtailed by CT. A maximum case number is reached within the same time as in the scenario without CT, which is, however, more than 10-fold lowered (Figure 3C). Afterwards, case numbers reach a plateau phase with *R_eff_* close to one. In this phase, they start decreasing slowly due to a decreasing ratio *S*/*N*. For indirect transmission, the case numbers are similarly reduced, reach a plateau as well, but still slowly increase after 20 weeks (Figure 3D).

**Figure 4:**
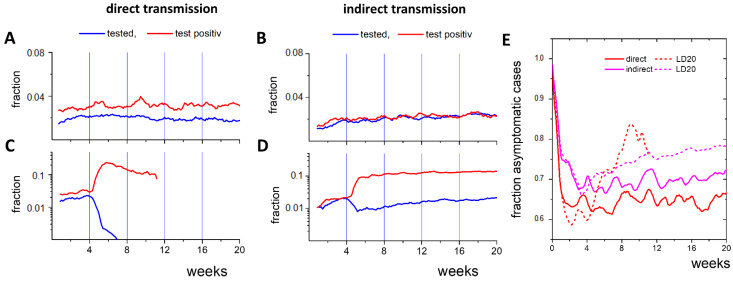
CT properties for direct and indirect transmission. (**A**,**B**) Fraction of agents tested per day and fraction of the positive tests. (**C**,**D**) LD20 at *t* = 4 weeks results in a strong increase of the test positive fraction. The fraction of tested agents falls below 0.001 two weeks after the lockdown start. (**E**) CT affects the fraction of asymptomatic cases among the undetected cases. Under LD20, this fraction increases. Results are averages over 10 simulations and 7 days.

In both scenarios, about 2% of all agents are tested every day (Figure 4A,B); i.e., about 120 contact agents per detected case. As about 0.2% of all untested agents are infected (Figure 3C,D: about 30 out of 16,000), 1% of these tests would be positive when including the index case. In fact, about 3% and 2% are infected for direct and indirect transmission, respectively. Thus, infected cases are enriched in the tested contacts, making CT efficient. The results suggest that effective CT provides an option to reach high levels of immunity under controlled conditions. However, the test effort is significant. A strategy to reduce the effort is to ignore ‘short term contacts’, i.e., contacts shorter than 15 min. In our simulations, 75% of the contacts are short term (Figure S2B, Supplementary Materials). However, ignoring them reduces the number of required tests by 25% only, as multiple contacts (7–9 on average) occur between agents; 1.5% of the population still has to be tested daily. Thus, the large quantities of tests required, particularly at high incidence, suggest the necessity of additional measures.

Next, we studied LD scenarios for both transmission types during active CT. We initialized an LD20 about 4 weeks after simulation start. During LD20, the numbers of contacted individuals reduced by about 89% compared to the reference, which is similar to observations during strong LDs in the COVID-19 epidemics (Wuhan 86%, Shanghai 89% [35]). In contrast, contact with neighbors intensifies. In the case of 100% direct transmission, the LD is efficient (*R_eff_* < 1). After a short delay of about 1 week, the number of cases decreases, and after 8 weeks, it reaches 1/3 of the initial value (Figure 3E). In the case of 100% indirect transmission, the LD is counterproductive for CT efficacy (*R_eff_* > 1). After a similar delay, the incidence begins to increase faster than without the LD, and after 8 weeks, it reaches more than 5 times the value at LD initiation (Figure 3F).

In both scenarios, the LD results in a strong increase of the number of positive tests (Figure 4C,D). This shows that intense contact between neighbors, i.e., agents close to base positions, during the LD increases transmission. For direct transmission, about 57% of the contacts are still short-term. However, they are responsible for only 5% of the directly transmitted infections (Figure S2B, Supplementary Materials). Direct transmission preferentially occurs via long-term contact, which typically occurs in households (Figure S2C, Supplementary Materials). Thus, it can be controlled through CT. In the case of indirect transmission, a higher local transmission occurs during the LD as well. In parallel, the probability that the agent that contaminated the area responsible for the observed infection does not contact the traced agent increases (72% instead of 20%). Asymptomatic infected agents become harder to identify and thus, transmission becomes more difficult to control. Notably, the fraction of asymptomatic cases in the untested population increases during LD for both transmission types (Figure 4E). In the case of direct transmission, this relates to a few remaining cases, while in the case of indirect transmission, these cases drive the epidemic.

**Limitations of non-pharmaceutical interventions.** So far, we have provided a picture of the epidemic based on the numbers of infected agents. In order to obtain a value that is independent of the population size, epidemics are typically characterized by the 7-day incidence of observed cases IN7_ob._ IN7_ob_ is lower for *F_A_* > 0 than the total incidence IN7_to_ (here, new cases per 100,000 individuals within 7 days). Without CT, IN7_ob_ is the number of new symptomatic cases within 7 days (IN7_S_). With CT, IN7_ob_ is the number of newly detected cases in this period (IN7_CT_, Figure S3, Supplementary Materials). Both values are strictly correlated with IN7_to_ and do correctly characterize spreading dynamics.

**Figure 5:**
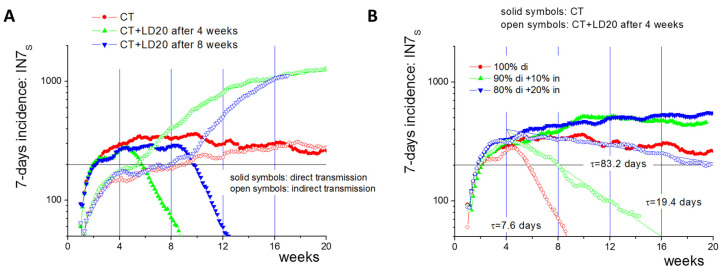
Simulated 7-day incidence IN7_s_ for different transmission routes (averages over 10 simulations). (**A**) In the case of an LD20, the incidence decays exponentially towards 50 within 4 weeks for direct transmission; for indirect transmission it rises to values above 1000. (**B**) A 10% and 20% contribution of indirect transmission increases the plateau level incidence by a factor less than 2. Under LD20, it increases the IN7_s_ half-life-time by a factor of about 2.5 and 11, respectively. Lines describe an exponential decay from level 400 with half-life-time τ.

Calculating IN7_s_ (or IN7_CT_) links our simulation results for small populations (*N* = 16,000) to the spreading of an epidemic in entire countries. LDs are often initialized depending on threshold values of IN7 [36]. In the case of direct transmission, a mobility LD20 can reduce IN7_S_ within 4 weeks from values above 200 to below 50 (Figure 5A). Contrarily, the same LD increases IN7_S_ to values above 1000 in cases of indirect transmission. For the chosen parameter set, the IN7_s_ half-life-time τ in the LD20 under direct transmission is about 7.6 days (Figure 5B). As both types of transmission might occur together, we combined them in our simulations, keeping the combined transmission rate at 0.2/day. Recent studies report a low infection risk from exposure to contaminated surfaces [2,37]. Thus, we considered direct transmission as the dominant transmission route. However, we found that indirect transmission strongly affects epidemic spread under LD20, even if the related risk of infection is an order of magnitude smaller than that of direct transmission. For a 10% fraction of indirect transmission (applying 0.1 *β_in_* and 0.9 *β_di_*), τ nearly triples to about 19.4 days. A fraction above 20% indirect transmission renders the LD20 inefficient (Figure 5B). Thus, an increasing share of indirect transmission reduces the impact of mobility LDs. Under certain circumstances, it can even lead to increasing numbers of infections.

One might expect that rather ‘soft’ LDs are more appropriate in the case of multiple transmission routes; first simulation results applying our model support this thesis. However, the advantage of a softer LD, i.e., the higher individual liberty, comes with a longer LD period and additional weeks of high incidence (Figure S4, Text S7, Supplementary Materials). Our results suggest that even in the case of small fractions of indirect transmission, one should give priority to reducing the number of contacts (similar to reducing the number of agents, Figure 2B) and not on reducing all agents’ daily mobility. Such physical distancing avoids increased transmission between neighbors as seen during mobility LDs, i.e., the fraction of positive tested agents monotonously decreases under the measure (Figure S4, Text S7, Supplementary Materials).

Throughout our study, we worked with a fraction of asymptomatic cases *F_A_* = 0.5 which estimates the fraction of undetected cases. In fact, this value limits the impact of CT. For increasing values of F_A_, the incidence of the plateau phase increases (Figure S5, Supplementary Materials). This can be understood as follows: With increasing *F_A_*, the transmission rate increases, and a higher number of infected agents has to be identified by CT to reach the plateau phase with an effective reproduction number *R_eff_* equal to one. For fixed numbers of contacts, this requires a higher incidence among them. The increasing incidence comes with increasing numbers of symptomatic cases. This compensates for the decrease in this number at increasing *F_A_*. IN7_S_, however, reaches a global maximum of around *F_A_* = 3/4 (Figure S5, Supplementary Materials). Thus, approaching *F_A_* = 3/4, the number of symptomatic cases becomes too small to control the epidemic. Accordingly, CT becomes inefficient. To keep it efficient, *F_A_* can be reduced by applying additional test strategies. However, this further increases the overall test effort.

## 4. Discussion

Here, we demonstrate that the efficacy of CT in controlling epidemic spread strongly depends on the fraction of direct and indirect transmission. Theoretically, CT can fully control a slightly overcritical epidemic (*R_eff_* slightly above 1). However, in real settings, its effect is smaller due to time delays in reporting, limited test efficacy, etc., and the incidence at which control is obtained might exhaust medical resources and thus lead to the implementation of additional measures such as lockdowns. However, not every measure is effective under a given condition. We simulated scenarios without vaccination efforts. While these efforts are the most promising, they are not always available and might be cost intensive. Moreover, waning effects can impair effectiveness of such strategies [38], highlighting the importance of CT even if vaccination can be applied. Including waning immunity following vaccination and infection in agent-based models thus represents a next step in validating the efficacy of CT, thereby extending existing model approaches [39,40].

Generally, reduced mobility in LDs leads to longer contact times, increasing the probability of infections of neighbors. Indeed, a higher infection risk in confined spaces has been noticed regarding the COVID-19 epidemic [41]. CT allows controlling this increase in cases of direct transmission, as infected agents can be identified efficiently. In the case of indirect transmission, the LD impedes identification of asymptomatic cases, and even a small fraction of indirect transmission may counteract disease control. Here, efficient control would require tracing agent position. This allows linking asymptomatic infected agents that contaminate an area with those who visit the area later on and become infected. Several countries, in particular in East Asia, implemented such measures during the COVID-19 epidemic early in 2020, which might be one reason for their successful control of spreading of the Wuhan and alpha variants. However, the impact of these interventions on individual liberty is significant. Moreover, the epidemic in Vietnam in July 2021 indicates that this approach becomes less effective for more infectious variants [42]. Higher fractions of asymptomatic (undetectable) cases F_A_ might explain that observation.

Although calculated for an abstract epidemic, similar effects of lockdowns were observed for the COVID-19 epidemic. In summer of 2020, following a mobility LD, a half-life-time of the incidence of about two weeks was observed in several European countries including Germany. During the second German LD in winter of 2020, this time was about four weeks (data: Johns Hopkins University). Thereby, survival of the virus indoors should be comparable. Whether this increase is related to higher indirect transmission because of strictly reduced mobility remains open. In any case, our results point to the particular importance of hygiene in the course of mobility LDs. The frequent cleaning of non-adhesive surfaces that are potentially contaminated either by direct contact or deposition of virus via aerosols should be mandatory [43]. In households, disinfection or wearing masks are similarly effective in reducing secondary transmission of SARS-COV-2 [44]. Under unrestricted mobility, these efforts become less important [45].

The described effects of indirect transmission require a sufficient half-life-time of the virus on high-touch surfaces. We have assumed a half-life-time for virus survival of 15h, a value at the upper limit suggested by several studies for non-adhesive surfaces [7]. However, it has been shown that protein in contact with the virus, as typically present, e.g., in sputum, can strongly increase the half-life-time to several days [46]. In face masks, viable virus has been detected even after 3 weeks [8]. The conditions actually required for an infection via indirect transmission are, as for direct transmission, largely unknown, making more realistic modeling impractical. We assumed a linear proportionality between the virus load and infection risk in agreement with assumptions of other studies for small virus concentrations [25]. A more sophisticated approach would require a measured dose-response curve [47], which is currently not available.

Testing, regardless of whether performed in the course of CT or other strategies such as the serial testing of subpopulations, is fundamental in controlling an epidemic, though the regulatory effort can be significant. In our simulation, ideal CT requires testing about 2% of the population daily, a value hard to reach on a longer time scale by imposed testing. Such control rates require combining self-imposed prevention measures and government imposed testing as suggested early by model studies [48]. The latter might comprise regular testing in schools, for example.

The actual effort highly depends on the fraction *F_A_* of cases that would not be detected without testing. Many of these cases might refer to individuals with existing cross-immunity, comprising up to 30% of the population [49,50], and vaccinated individuals after a waning period [51]. One might expect that these individuals are also less infectious and thus do not drive spreading. However, there is strong evidence against this assumption [52], although virus clearance seems to be faster in vaccinated individuals [53]. Values estimated for *F_A_* in an epidemic depend on the method applied and the spreading dynamics [54]. The latter is also seen in our simulations, reaching values found in hotspots (>0.7) under LD for an intrinsic value of the epidemic of *F_A_* = 0.5. Thus, LD values of *F_A_* might be overestimations. This may at least in part explain observed local differences.

## 5. Conclusions

Agent-based simulations of epidemic spread allow the consideration of agent mobility. This mobility not only affects spreading dynamics, but it also enables a straightforward simulation of CT. Here, we show that CT alone can stabilize an epidemic in the case of moderate fractions of asymptomatic cases. However, its efficacy changes under mobility LDs. LDs improve CT efficiency in cases of exclusive direct transmission, while even a small fraction of indirect transmission can be sufficient to impede CT efficacy and to accelerate spreading under such conditions. Thus, detailed knowledge regarding transmission routes is crucial to determine efficient non-pharmaceutical intervention strategies.

## Data Availability

Not applicable.

## Supplementary Materials

The following supporting information can be downloaded at: https://www.mdpi.com/article/10.3390/ijerph192114011/s1, Text S1: Estimation of epidemic parameters from public data, Figure S1: Plots of daily and recovered cases; Text S2: Program design and performance; Text S3: Properties of the epidemic, Figure S2: Time delay for recovery and contact properties; Text S4: R0 estimations for the jump model; Text S5: Estimation of epidemic maximum considering symptomatic and asymptomatic cases; Text S6: 7-day incidence, Figure S3: Estimates of the 7-day incidence; Text S7: Alternative strategies to control the epidemic, Figure S4: Different measures in the presence of multiple transmission routes; Text S8: Limitations for CT efficiency, Figure S5: Fraction of asymptomatic cases defines the incidence in the plateau phase. Figure S6: Reproduction of Figure 3 as greyscale readable. Epidemic spread for direct and indirect transmission.
