# Supplementary material for: Indirect Virus Transmission via Fomites Can Counteract Lock-Down Effectiveness
Source: Int J Environ Res Public Health. 2022 Oct 27;19(21):14011. doi: 10.3390/ijerph192114011 (PMC9658534; doi:10.3390/ijerph192114011)
Supplement: Supplementary file 1 [file ijerph-19-14011-s001.zip › ijerph-1916286-supplementary.pdf]

## Supplementary Materials:

### Text S1: Estimation of epidemic parameters from public data.

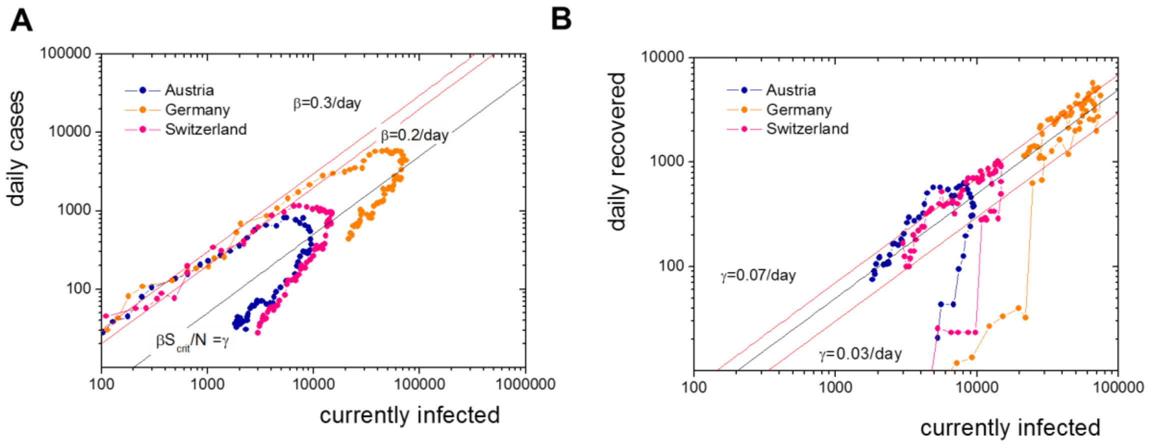


**Figure S1:** Plots of daily cases (A) and daily recovered cases (B) against the number of currently infected cases during the first wave of SARS-CoV-2 in 2020 in the countries indicated. Lines refer to selected values of $\beta$ and $\gamma$, respectively. In our study, we used $\beta$ = 0.25 and $\gamma$ = 0.05. Data provided by the Johns Hopkins University (https://systems.jhu.edu/research/public-health/ncov ).

### Text S2: Program design and performance

The purpose and scope of the model have been described in the Introduction; variables and initialization of the model in the Methods section. Here, we provide a short process overview.

The program calculates the state and position of the N=S+E+I+R agents at time t+dt based on its state at time t in the following steps:

1) Each agent that occupies state E since $T_E$ days switches into state $I_a$, if an evenly distributed random number ∈ [0, 1] generated for it is smaller than $F_A$. Otherwise, it switches into state $I_s$.
2) Each agent that occupies since $T_I$ days or longer state $I_s$ or $I_a$ switches into state R, i.e. recovers, if an evenly distributed random number ∈ [0, 1] generated for it is smaller than $\Gamma$dt. Agents that recover in quarantine return to their base position.
3) Agents that are not in quarantine get moved.
4) In cases of indirect transmission, the state of contamination of all interaction points is recalculated.
5) Agents that occupy since $T_N$-$T_E$ days state $I_s$ become symptomatic and are quarantined, i.e. become non-interacting. Their contacts within the last week are tested and test positive contacts (∈ I) become immediate quarantined as well.
6) For agents in state S, an individual decision is made about a potential infection (see text). All infected agents switch into state E.
7) All agents return to their base position, if they have moved a time period $t_{ret}$.
8) The time is set to: t=t+dt and the program returns to 1).

According to step 1-8, random decisions are implemented on the level of individual agents regarding i) the infection type, ii) the recovery time of asymptomatic agents, and iii) movement. Parameters are set according to published data of the Covid-19 epidemics (see: Tab. 1). Beside the parameters, the model requires no further input data. Details of agent movement and interactions, i.e. virus transmission, are explained in the Methods section. The model results have been compared to analytical solutions of the SEIR model to ensure correct implementation and to decide about reference agent density and mobility (see text). On average, a simulated week for N=16000 agents costs 5.4 [1.4] h runtime at a Bull/Atos cluster of the TU Dresden Center for High Performance Computing (HPC).

## Text S3: Properties of the epidemic.

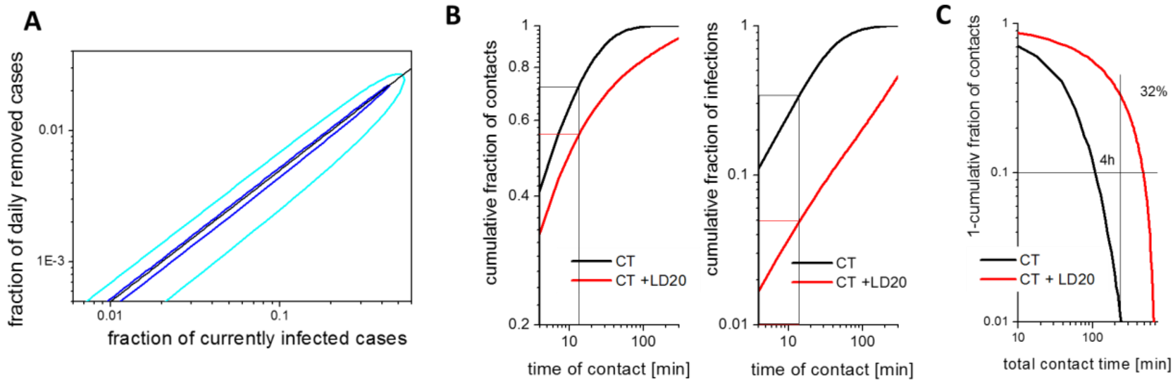


**Figure S2: Time delay for recovery and contact properties.** A) A time delay to leave the infected state and to reach the recovered state separates the SEIR branches for daily recovered cases. The lower branch refers to increasing and the upper branch to decreasing numbers of infected cases. Delay: 1 day (blue), 5 days (cyan). A similar effect is seen in SARS-CoV-2 data (Figure S1B). The straight black line is the result for immediate recovery with rate k=0.05/day. B) Cumulative fraction of contacts (left) and infections (right) during contacts of length t for reference mobility and LD20 with CT (cut point: t=300min). C) Cumulative fraction of contacts for the total contact time between agents. About one third of the contacts during LD20 occurs at total contact times (>4h) not reached at reference conditions.

## Text S4: $R_0$ estimations for the jump model

We start estimating $R_0$ for the jump model without quarantine. Our estimates hold in the limit of large N: We fix a random infected agent $i_0$ inserted into the system at time 1 and denote the random time the person is infectious T. T is measured in units of $\Delta t$ for position update. For a susceptible agent i $\in$ (1,…,N-1), we denote by $X_i(\tau)$ the random variable $\in$ [0, 1] encoding the event that agent i is at time $1 \leq \tau \leq T$ infected by $i_0$. $X_i(\tau) = 0$ means that agent i did not get infected at time $\tau$ by $i_0$. Note that we count infections with multiplicities and hence $X_i(\tau)$ are iid random variables. The individual (random) reproduction number $R(i_0)$ of individual $i_0$ is given by: $R(i_0) = \sum_{\tau=1}^{T} \sum_{i=1}^{N-1} X_i(\tau)$.

We are interested in the expectation of $R(i_0)$, which is the reproduction number $R_0$. Applying Wald's theorem, we get:

$$R_0 = E(T)(N-1)E\big(X_1(1)\big), \text{ with: } E(T) = \frac{T_I + 1/\Gamma}{\Delta t} \text{ and: } E\big(X_1(1)\big) = \frac{\Delta t \beta_{di} 4\pi r_{in}^2}{A}, \qquad \text{Equ. S1}$$

where A is the system area and $r_{in}$ the contact radius. For the reference value $N_{ref}$ = 16000 and the parameters chosen from the Table 1, we get: $R_0 \approx 10.05$. For the jump scenario with quarantine of symptomatic cases ($F_A$ = ½, i.e. $E(T) = \frac{(T_I + 1/\Gamma + T_N - T_E)}{2\Delta t}$) one gets: $R_0 \approx 5.63$.

## Text S5: Estimation of epidemic maximum considering symptomatic and asymptomatic cases.

For the weighted sum of exposed (*E*) and infected (*I*=*I*$_s$+*I*$_a$) cases: $= \frac{1}{2}\left(\frac{1}{\gamma_s} + \frac{1}{\gamma_a}\right) E + \frac{1}{\gamma_s} I_s + \frac{1}{\gamma_a} I_a$ ,

we have: $$\frac{dA}{dt} = \frac{1}{2}\left(\frac{1}{\gamma_s} + \frac{1}{\gamma_a}\right) \beta S \frac{I_s + I_a}{N} - \frac{I_s + I_a}{N} = \left[\frac{1}{2}\left(R_0^s + R_0^a\right) S/N - 1\right](I_s + I_a) , \qquad \text{Equ. S2}$$

where $\beta$ is the infection rate and $\gamma_s$ and $\gamma_a$ are the recovery rates of symptomatic and asymptomatic cases, respectively. Accordingly, for the critical maximum condition yields: $S_{crit} = \frac{2N}{R_0^s + R_0^a} = N/R_0^m$.

With $B = \beta A$, we define the invariant: $\frac{1}{2}(R_0^s + R_0^a)\, S/N + B/N - ln\,(S/N)$.

For the maximum $S_{crit}$, we get: $B/N = R_0^m - 1 - ln(R_0^m)$ and for $F_A$=1/2, i.e. $I_s = I_a = I/2$,

$$B/N = \frac{1}{2}(R_0^s + R_0^a)\, E/N + \left(\frac{R_0^s I}{2} + \frac{R_0^a I}{2}\right)/N = R_0^m\, (E + I)/N\ .$$

Thus, we get: $J_{max}/N = max\,(E + I_s + I_a)/N = 1 - \left(1 + ln\,(R_0^m)\right)/R_0^m$. Equ. S3

**Text S6: 7-day incidence**

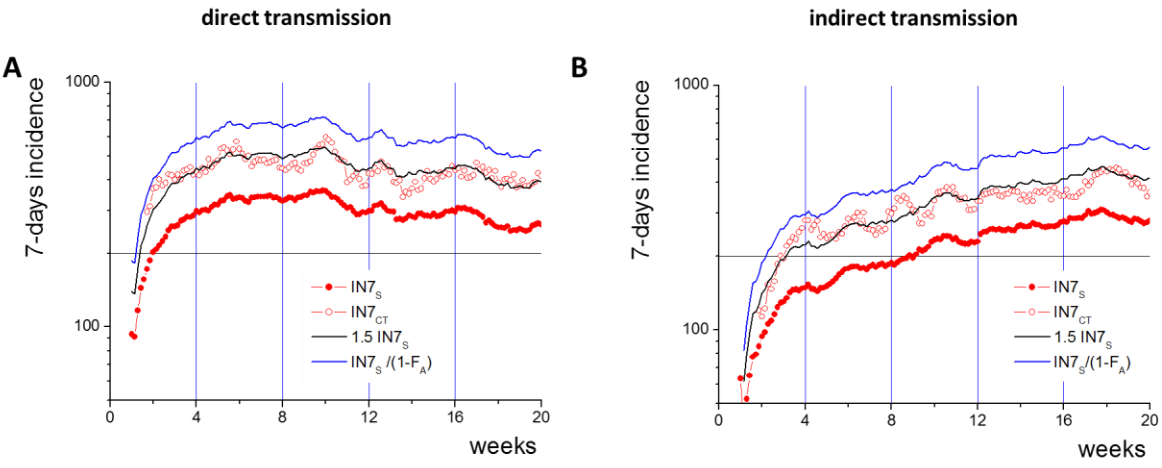


**Figure S3: Estimates of the 7-day incidence.** $IN7_{ob}$ at $F_A$=0.5. The results are averages of 10 simulations. $IN7_{ob}$ is calculated based either on all symptomatic cases ($IN7_S$) or on all symptomatic and all detected asymptomatic cases ($IN7_{CT}$). The test effort increases the incidence for $F_A$=0.5 by a factor of about 1.5 (black line). The time course remains similar. The total incidence, $IN7_{to}$ (blue line), is given by: $IN7_S/(1-F_A)$.

**Text S7: Alternative strategies to control the epidemic.**

At 10% indirect transmission, a 'softer' LD reducing the mobility to 40% of the reference value (LD40) induces a similar decay as the 'strong' LD20 (Figure S4A). However, this decay starts about 3 weeks later than that of the 'strong' LD. Thus, the advantage of the softer LD comes along with a longer LD period and an additional 3 weeks of high incidence.
As an alternative measure, we simulated improved physical 'distancing'. This method reduces the number of contacts as follows: We created a network of acquaintance among the agents, where each agent $a_i$ knows only a specific subpopulation $P(a_i)$. Afterwards, we run simulations where all agents strictly avoid all contacts to agents not being part of $P(a_i)$. Without reducing mobility (LD100), a contact reduction to a third (ID33) induces a steep decrease of the 7-day incidence in the first two weeks (Figure S4B). The long-term decay in the presence of 10% indirect transmission is similar to a mobility LD20 but occurs on a lower absolute value. The fraction of positively tested agents monotonously decreases under the measure (Figure S4C).

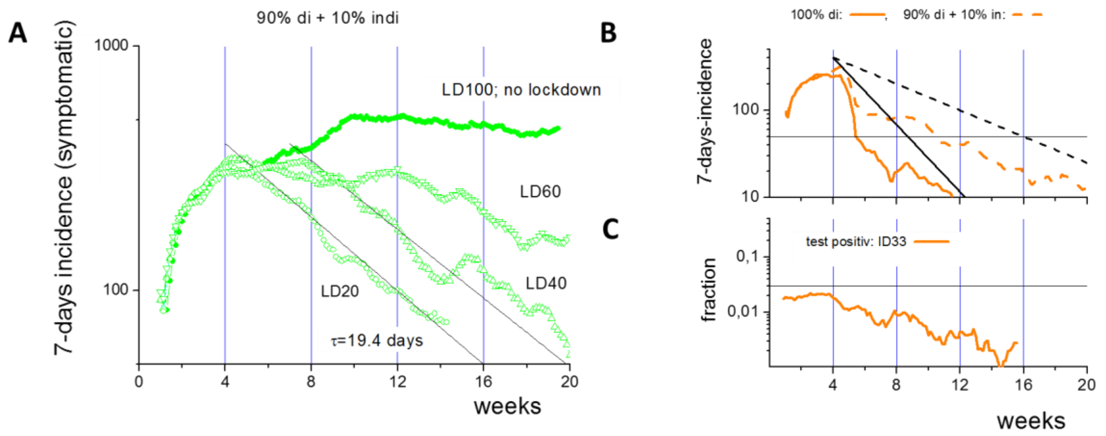


**Figure S4: Different measures in the presence of multiple transmission routes.** A) A similar decay of the 7-day incidence is observed for a strong LD20 and a softer LD40. The onset of the decay for LD40 occurs later. Lines describe an exponential decay from incidence level 400 and decay rate $\tau$ starting at t=4 and 7 weeks. B-C) Moderate distancing can be as efficient as a strong reduction of mobility. B) Faster reduction of the 7-day incidence under improved distancing ID33 (orange) compared to a LD20 (straight lines from Fig. 5B) in the first two weeks for both 100% direct transmission (solid line) and with 10% indirect transmission (dashed lines). The long-term decay for ID33 is similar to LD20. Both measures are applied starting at t=4 weeks. C) The fraction of positively tested agents (100% direct transmission) continuously decreases under distancing (ID33).

## Text S8: Limitations for CT efficiency

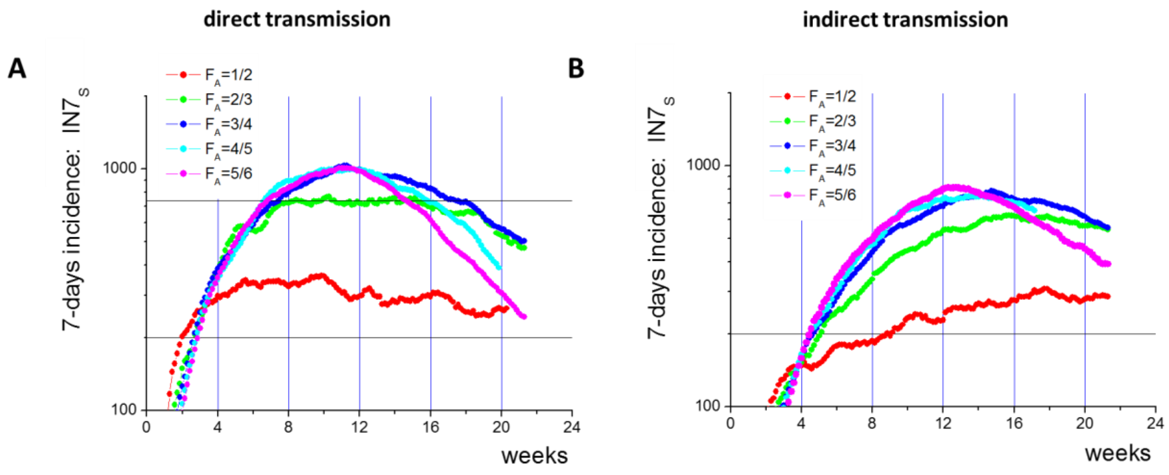


**Figure S5: Fraction of asymptomatic cases defines the incidence in the plateau phase.** IN7$_S$ under CT for different values of $F_A$. The results are averages of 10 simulations. Increasing the fraction of asymptomatic cases, $F_A$, increases the incidence in the plateau phase. Above $F_A$=2/3, the plateau phase starts vanishing. IN7$_S$ reaches a maximum at about $F_A$=3/4 after 11 and 15 weeks for 100% direct and indirect transmission, respectively.

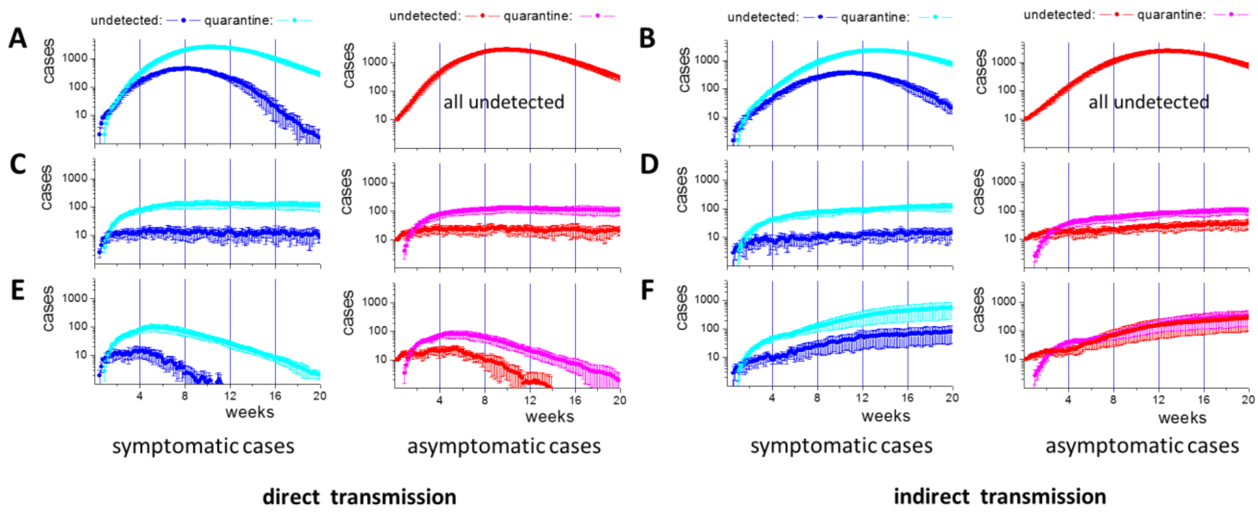


**Reproduced Fig. 3** (greyscale readable)

**Figure S6. Reproduction of Figure 3 as greyscale readable. Epidemic spread for direct and indirect transmission.** Time series of case numbers for controlled spreading without CT (**A**,**B**), with CT (**C**,**D**) and for LD after 4 weeks in parallel to CT (**E**,**F**). Case numbers are shown for detected and undetected symptomatic and asymptomatic cases (averages over 10 simulations, errors: sd). Without CT, case numbers peak between 8 and 13 weeks. With CT, they stabilize after short time at a much lower level. An LD20 is efficient for direct ($R_{eff} < 1$) but counterproductive ($R_{eff} > 1$) for indirect transmission.
